# Ordered ZnO/Ni Hollow Microsphere Arrays as Anode Materials for Lithium Ion Batteries

**DOI:** 10.3390/ma12071193

**Published:** 2019-04-11

**Authors:** Shijie Shen, Wenwu Zhong, Xiaohua Huang, Yan Lin, Tianle Wang

**Affiliations:** Department of Materials Engineering, Taizhou University, Taizhou 318000, China; shensj@tzc.edu.cn (S.S.); tianmenwenwu@163.com (W.Z.); linyan@tzc.edu.cn (Y.L.); wtl0203@tzc.edu.cn (T.W.)

**Keywords:** Zinc oxide, hollow microsphere, nanocomposite, anode materials, lithium ion battery

## Abstract

Well-designed nanostructures are very important for the electrochemical performance of lithium-ion electrode materials. In order to improve the electrochemical performance of ZnO-based anode materials, ZnO/Ni composite film, assembled by ordered hollow microsphere arrays, is designed and fabricated by means of magnetron sputtering technique using a colloidal crystal template composed of a monolayer of ordered polystyrene (PS) microspheres. The ordered hollow microsphere structure as well as the constituent Ni component of the ZnO/Ni film show major advantages of homogenizing electrode reactions, enhancing electrode reaction kinetics and accommodating volume change of active materials, so they can reduce electrode polarization and stabilize electrode structure. Consequently, the resulting ordered ZnO/Ni hollow microspheres arrays deliver an initial charge capacity of 685 mAh g^−1^, an initial coulombic efficiency of 68%, and a capacity retention rate of 69% after 100 cycles, all of which are higher than those of the pure ZnO film. These results show progress in developing more stable ZnO-based anode materials for lithium ion batteries.

## 1. Introduction

Transition-metal oxides are important materials for energy storage and conversion [1,2]. Zinc oxide, as a typical transition-metal oxide, is a promising anode material for lithium-ion batteries due to its high theoretical capacity of 988 mAh g^−1^ that is ~2.7 times that of traditional graphite [3,4]. However, the research on ZnO-based anode materials is far less than other transition-metal oxides due to its much poorer actual electrochemical performance. For example, ZnO often experiences dramatic capacity decrease, even to meaningless values, just in the early stages of the cycling process. Designing nanostructures and forming composites are common ways to overcome this problem and they are often used in combination. Over the past decade, plenty of ZnO-based composites have been prepared by introducing metals and/or carbon [5,6,7], and meanwhile, they have been designed as various nanostructures, for example, porous [8,9], hollow [10], spherical [11], core/shell [12], and yolk/shell [13].

Unlike other transition-metal oxides, the reduction product of ZnO by Li, i.e., metallic Zn, is still electrochemically active towards lithium. Therefore, theoretically, the lithium-storage mechanism of ZnO includes two steps, which are, the conversion reaction (ZnO + 2Li ⇌ Zn + Li_2_O) as well as the alloying/dealloying reaction (Zn + Li ⇌ LiZn) [3,4]. However, these two reactions are usually partially reversible, and meanwhile, they both cause large electrode volume changes, which are responsible for the extremely poor electrochemical performance of ZnO anode materials. Therefore, rational designing the component and structure of electrode materials to increase the utilization rate and accommodate the volume change at the same time is an important approach to achieve enhanced electrochemical performance.

Herein, an unconventional ZnO/Ni composite electrode with a nanostructure of ordered hollow microsphere arrays is designed and fabricated. It is expected that the ordered ZnO/Ni hollow microsphere arrays have better abilities of homogenizing electrode reactions, enhancing electrode reaction kinetics and accommodating electrode volume change.

## 2. Materials and Methods

### 2.1. Sample Preparation

Ordered ZnO/Ni hollow microsphere arrays were prepared by magnetron sputtering using a colloidal crystal template composed of a monolayer of ordered polystyrene (PS) microspheres. Commercial dispersion of PS microspheres with a diameter of 1 μm was diluted by a mixture of ethanol and distilled water to obtain a monolayer of ordered PS microspheres at the liquid/gas interface. The monolayer was picked up by a polished stainless-steel plate and transferred to its surface. The deposition of ZnO and Ni on the PS template was proceeded by magnetron sputtering (DE500, DE Technology, Beijing, China). ZnO and Ni targets were fixed at 20 cm above the substrate holder. After the chamber was pumped to a base pressure of 0.5 μTorr, high purity argon (99.999%) was introduced as working gas and the pressure was maintained at 8 mTorr. ZnO was deposited first for 1000 s under radio-frequency power of 50 W, and then Ni was deposited for 500 s under direct-current power of 50 W. The resulting sample was finally annealed at 300 °C for 2 h in vacuum to remove the PS template. For comparison, pure ZnO film was prepared by radio-frequency magnetron sputtering under 50 W for 10 h on a bare stainless-steel substrate.

### 2.2. Materials Characterizations

Ordered ZnO/Ni hollow microsphere arrays were characterized by means of scanning electron microscopy (SEM, S–4800, Hitachi, Tokyo, Japan) and transmission electron microscopy (TEM, Tecnai G2 F20, FEI, Hillsboro, OR, USA). The mass of the components in the film was weighed by a high-precision balance in a simultaneous thermal analyzer (STA 449 F3, Netzsch, Selb, Germany).

### 2.3. Electrochemical Measurements

CR2025 cells were used for electrochemical tests, which were assembled in an argon-filled glove box using the film as working electrode and lithium foil as counter electrode. The electrolyte was 1 M LiPF_6_ dissolved in a mixture of ethylene carbonate (EC) and dimethyl carbonate (DMC) with a volume ratio of 1:1.

Galvanostatic discharge–charge tests of cells were performed on a battery test system (CT2001A, LAND, Wuhan, China) using different current densities from 0.1 to 2.0 A g^−1^ in the voltage range of 0.02–3 V. Cyclic voltammetry (CV) tests were carried out on an electrochemical workstation (PGSTAT302N, Autolab, Utrecht, The Netherlands) at a scan rate of 0.1 mV s^−1^ between 0 and 3 V.

## 3. Results and Discussion

**Figure 1:**
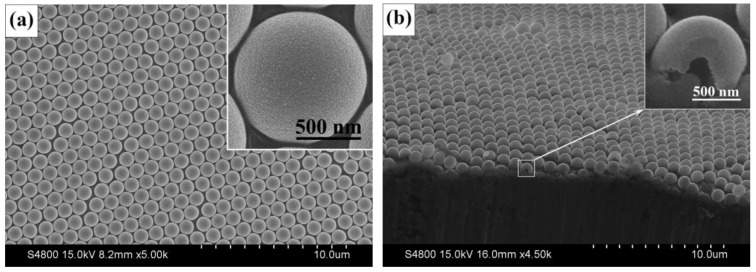
SEM images of ordered ZnO/Ni hollow microsphere arrays, (**a**) top-view image and (**b**) cross-sectional image.

SEM images of ZnO/Ni film are shown in Figure 1. The film is composed of microspheres with almost the same diameter (Figure 1a). These microspheres are close-packed and periodically assembled as a single-layer ordered structure. The magnified image of a single microsphere indicates a diameter of about 1 μm. The cross-sectional image (Figure 1b) of the electrode confirms the monolayer structure of the PS microsphere stacking. It is known from a cracked microsphere that it has a hollow structure.

**Figure 2:**
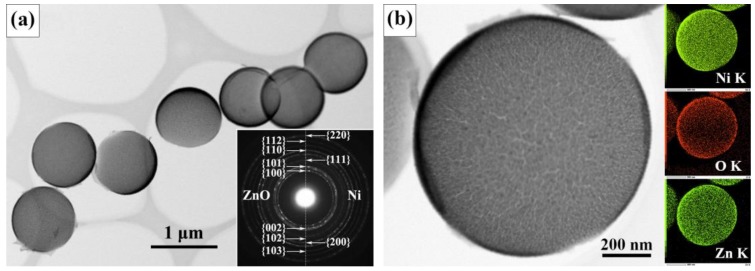
TEM results of ZnO/Ni hollow microspheres, (**a**) the low-magnification image and the corresponding SAED pattern, (**b**) the image of a single microsphere and the corresponding EDS mapping of Zn, O, and Ni elements.

TEM results of the microspheres are presented in Figure 2. The low-magnification image (Figure 2a) shows typical morphology of hollow spheres. Selected area electron diffraction (SAED) pattern displays two sets of diffraction rings that can be assigned to ZnO and Ni, respectively, indicative of the polycrystalline nature of each component. The image of a single hollow microsphere (Figure 2b) shows that it is assembled by ultrafine nanoparticles. EDS mapping of Zn, O and Ni elements indicates that both ZnO and Ni are distributed uniformly.

According to the weight increments of the film, it can be determined that the average areal densities of ZnO and Ni components in the ZnO/Ni composite film are ~65 and ~40 μg cm^−2^, respectively.

**Figure 3:**
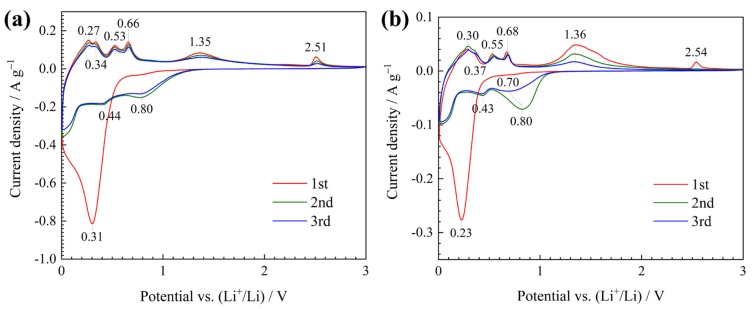
Cyclic voltammetry (CV) curves of (**a**) ZnO/Ni and (**b**) ZnO electrodes.

The electrochemical performances of ordered ZnO/Ni hollow microsphere arrays and pure ZnO film are compared under the same test conditions. CV curves tested at 0.1 mV s^−1^ are presented in Figure 3. Both two curves show only one cathodic peak at ~0.3 V with high intensity in the first scan, which is the common feature of all transition-metal oxides. This peak is related to the electrochemical reactions between ZnO and Li including the conversion reaction of ZnO to Zn and the further alloying reaction of Zn to LiZn, as well as other side reactions such as the formation of solid electrolyte interface (SEI) layer [14,15]. Apart from the first cathodic scan, the curves are quite similar in shape. The cathodic peak at ~0.4 V is related to the alloying reaction of Zn with Li. The first four low-potential anodic peaks below 0.7 V ascribe to the multi-step dealloying process of LiZn [16,17]. The last anodic peak at ~2.5 V corresponds to the conversion reaction of Zn back to ZnO [4,14,16]. The other pair of cathodic and anodic peaks, near 0.8 and 1.3 V, respectively, correspond to the partially reversible formation/decomposition of a ‘polymer/gel-like layer’ that is generated from side reactions occurring at the interface [2,18]. However, it is obvious that the CV curves of ZnO electrode show two differences compared with those of ZnO/Ni electrode. Firstly, its 2.5 V anodic peak appears only in the first cycle, which implies that the conversion reaction mechanism, i.e., Zn + Li_2_O → ZnO + 2Li, does not exist in the subsequent cycles, and this undoubtedly reduces the utilization of the active material and thus affects the reversible capacity. Secondly, the pair of peaks at 0.8 and 1.3 V, show higher but rapidly decreased intensities in the cycling process, which indicates that the ‘polymer/gel-like layer’ is quite electrochemically unstable in the early cycles.

**Figure 4:**
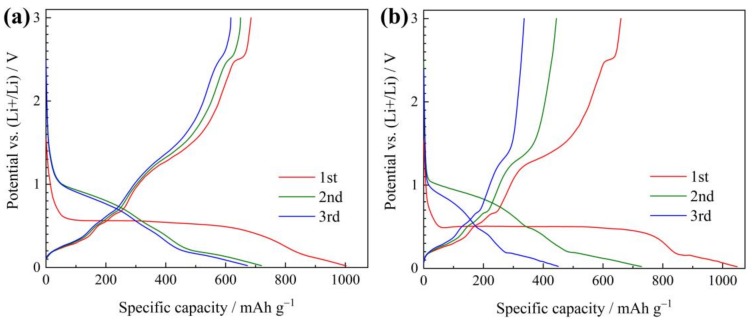
Galvanostatic discharge–charge curves of (**a**) ZnO/Ni and (**b**) ZnO electrodes.

Galvanostatic discharge–charge curves of the two electrodes tested at 0.1 A g^−1^ are compared in Figure 4. The potential of plateaus or slopes are almost consistent with those of the peaks in CV curves. For ZnO/Ni electrode (Figure 4a), it delivers an initial reversible capacity (charge capacity) of 685 mAh g^−1^ and an initial coulombic efficiency of 68%. For ZnO electrode (Figure 4b), these values are lower, only 660 mAh g^−1^ and 63%, respectively.

**Figure 5:**
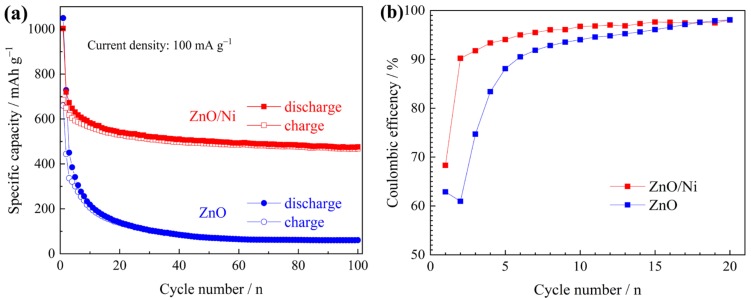
The evolution of (**a**) capacity and (**b**) coulombic efficiency during the cycling process of the two electrodes.

Cycling performances of the two electrodes tested at 0.1 A g^−1^ are shown in Figure 5. ZnO/Ni electrode exhibits much higher capacities during the cycling process, as shown in Figure 5a, and its charge capacity after 100 cycles is 470 mAh g^−1^, 69% of its initial value. In contrast, ZnO electrode fades very fast to the value below 100 mAh g^−1^ just after only 30 cycles, and the final capacity is as low as 60 mAh g^−1^. Figure 5b compares their coulombic efficiency in the early period. The higher coulombic efficiency of ZnO/Ni electrode indicates better reversibility of electrode reactions.

**Figure 6:**
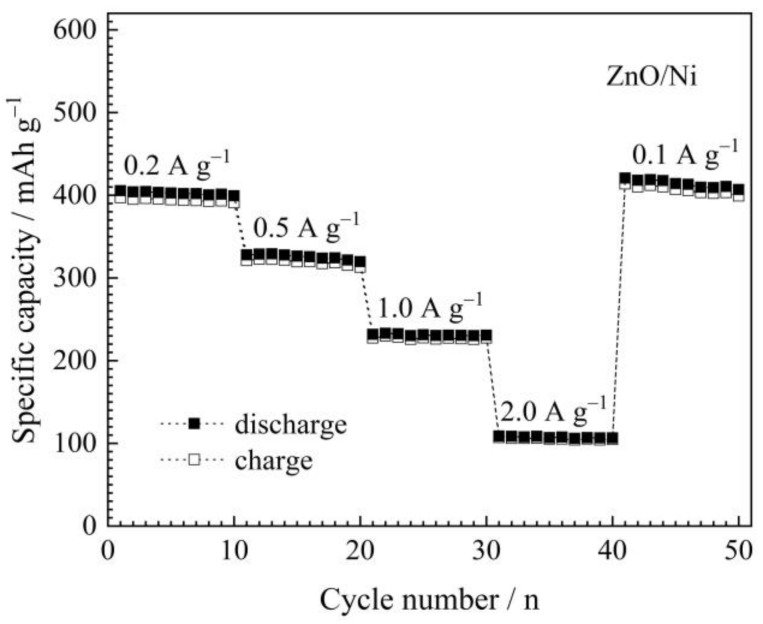
Rate capability of ZnO/Ni electrode tested at different current densities.

For the evaluation of rate capability, the above-mentioned ZnO/Ni electrode, which has been cycled for 100 times at 0.1 A g^−1^, continues to be tested at different current densities of 0.2, 0.5, 1.0, 2.0, and 0.1 A g^−1^ in turn, for 10 cycles at each stage, and the results are presented in Figure 6. It is obvious that the electrode still has some capacity of about 105 mAh g^−1^ at the highest current density of 2.0 A g^−1^, and finally, when the current density returns to the initial 0.1 A g^−1^, the capacities can rise back to about 405 mAh g^−1^.

**Figure 7:**
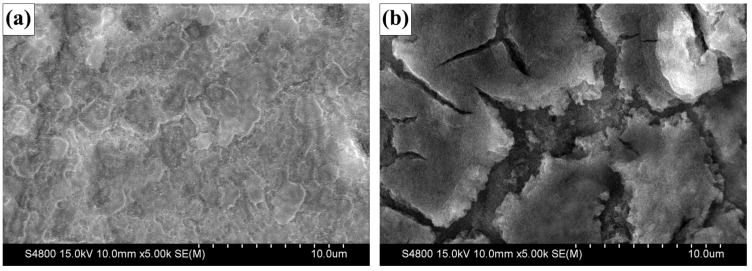
SEM images of (**a**) ZnO/Ni and (**b**) ZnO electrodes after 20 cycles.

Figure 7 compares the SEM images of the two electrodes after cycling. Both two electrodes are covered by the ‘polymer/gel-like layers’, which show some shrinkage under the long-time irradiation of electron beam. For ZnO/Ni electrode, the film is still continuously and evenly distributed, but for ZnO electrode, large cracks or even gullies can be observed clearly. Obviously, it can be concluded that the ZnO/Ni electrode has much better structure stability. The ordered hollow microsphere arrays are capable of uniformly absorbing the volume change of electrode materials, which is very advantageous for minimizing the internal stress and thus stabilizing the electrode structure. Conversely, for pure ZnO electrode, because of the lack of accommodation space for volume changes, the film fractures inevitably under the high stress. This not only causes active materials to peel off the electrode, resulting in fast capacity fading, but also creates new materials/electrolyte interfaces and forms new ‘polymer/gel-like layers’, resulting in decreased coulombic efficiency.

The nanostructure of ordered hollow microsphere arrays is also capable of reducing electrode polarization. Firstly, ordered-array arrangement ensures uniform distribution of active materials, which makes the electrochemical reactions proceed more homogeneously and completely, and this is beneficial to maximize the utilization of active materials. Secondly, the hollow microspheres offer a porous structure, which can enlarge reaction interface, reduce local current density and shorten charge transfer distance. In addition, the Ni component in the composite can also increase the electric conductivity. All these advantages are quite helpful to enhance the kinetics of electrode reaction, which is the key factor for the reversible capacity and rate capability.

## 4. Conclusions

In summary, ordered ZnO/Ni hollow microsphere arrays are successfully prepared by magnetron sputtering technique using a monolayer of polystyrene microspheres as the colloidal crystal template. These hollow microspheres have a diameter of 1 μm, and they are close-packed into an ordered-array structure. As anode materials for lithium-ion batteries, ZnO/Ni electrode delivers significantly enhanced electrochemical performance including reversible capacity, coulombic efficiency, and cycling stability as compared to pure ZnO electrode. The improvements are attributed to the ordered hollow microsphere array structure and the Ni component of the ZnO/Ni electrode, as they show great advantages of reducing electrode polarization and stabilizing electrode structure.
